# Patterns of empathy as embodied practice in clinical conversation—a musical dimension

**DOI:** 10.3389/fpsyg.2014.00349

**Published:** 2014-04-29

**Authors:** Michael B. Buchholz

**Affiliations:** Social Psychology, International Psychoanalytic UniversityBerlin, Germany

**Keywords:** empathy, embodiment, conversation analysis, psychotherapy, cognitive linguistics, alterocentrism, transcripts

## Abstract

Cognitive linguistics and conversation analysis (a) converge in the analysis of category bound activities and (b) in viewing thinking and talking as embodied activities. The first aim of this paper is to outline these powerful theories as useful tools for the analysis of enacting empathy. The second aim is to outline these theories as useful tools for the analysis of how empathy is co-enacted in clinical conversation documented in transcripts. Cognitive Linguistics and Conversation Analysis converge in detecting patterns of I-You-relationships with roots in early preverbal embodied protoconversation continuing to more symbolic conversational level. The paper proposes to describe this continuity of empathic conversation in *musical* metaphors like balance, rhythm and resonance. In a first section transcripts from therapeutic sessions are presented. In a second section linguistic and other research data are presented in order to bring empirical data to this new conception of how empathy can be understood, how it is done and how two participants cooperate to enact empathy. Ideas for further research are outlined.

## Introduction

All therapists with a clinical background share some of the following experiences: talking to a depressive patient you suddenly realize how your voice changes. You speak in a calm and soothing manner, you turn your voice down to a gentle mode. It is not that your voice changes into this depressive-response mode gradually. It is there right from the start. You see your patient for the first time, you see her or his eyes and you “know” how to respond. But this is not (static) “knowledge” you have from a handbook, it cannot be written down in teachable and learnable sentences. It just happens, it is a process of “knowing,” less from “knowledge.” You adopt yourself to something you sense and often it takes a long time to realize that you did.

The phenomenon is far-reaching. Being married to another therapist, I have very often witnessed the fact that by listening to one's partner responding to the client's first phone call, the bystanding partner can make a kind of diagnostic proposal just from listening to the voice of the partner answering the phone. More often than not this diagnostic assessment turns out to be correct. Therapists have a different way of talking with anxious clients from that with obsessive compulsive ones, they change pitch, volume and speech rhythm when they talk to a client with a personality disorder in a different manner to when engaging with someone who is depressive. Very often this adaptation in *resonance* remains beyond conscious awareness.

Galatzer-Levy (2009) reports another experience. He had a patient who never responded after his therapist had said something. He simply was silent for half a minute or so and when speaking he would change topic. So the therapist was never informed via a feed-back loop whether he had said something correct or not, nor whether he had even been heard! Sometimes the therapist even got to feeling uncertain whether he had actually spoken or not. After having endured this derealiziation mode for a certain time he made up his mind to address the pattern directly. And what did the patient say? “I admit and go on.” Perplexed, the therapist responded with something like “You admit and go on” and quickly the patient now answered “Yes, I admit and go on.” This little exchange of the same phrase had a *rhythm*, spoken with groove and swing—both suddenly felt compelled to laugh. So in a difficult dialog format suddenly a sort of warm cheerfulness emerged, bringing this therapy into a new mode. Not the meaning of the words, it was the bodily dance-like rhythm that moved the therapeutic pair to a new level.

Elizabeth Nutt Williams (2008, p. 140) reports that one evening she wanted to review a video-taped session that she remembered as vivid, full of quick verbal exchange. “I was stunned to see a low-key, slow, and fairly quiet one instead. I was struck by the vast difference between my experience and the recorded tape.” How can this *imbalance* be accounted for?

When in June 2013 at a research-conference at “International Psychoanalytic University” (IPU) in Berlin, Germany, I presented a CA (Schegloff, 2007a,b) of a psychoanalytic first interview exchange I discovered a rhythmic element in it. The experienced therapist gave an impulse by asking a confrontational question—and when the patient answered the therapist did not come up with the next question, but withdrew with a conversational continuer like “hm:h” which pragmatically means something like “go ahead.” So on the transcript one could see a certain kind of rhythm: one hard beat (the impulse of a question) followed by 3–4 soft beats like “hm:h,” calmly spoken. This way of beginning an initiative left room for the patient's own initiatives. One could not say that this pattern was planned, it emerged from the situation. It was a kind of *musical* rhythm that made the burden of being interviewed a little lighter. It was one of those impressive interviews, wherein a skilled therapist manages to talk with a patient never seen before about deep involvement in certain sexual topics without being either intrusive or seductive.

This kind of experience can best be described by *musical* metaphors like resonance, rhythm or balance. Since Freud it is well known that psychic experience cannot be conceptualized but in metaphors. But is it necessary to hold on to metaphors of “inner” mental (or cognitive) life and “outer” real world as so many contemporary theorists like to do? Potter and Edwards (2013) analyze the consequences of such a distinction. These metaphors guide many quarrels about “cognition” vs. “social cause” of traumatic experience, they stem from a Cartesian tradition of separating body from mind. Using new metaphors as guide may contribute to get a deeper understanding of embodied simulation, of conversation and of helpful interactions. This gradually growing conviction inspired me to look for the *musical* structure of talk-in-interaction (Malloch and Trevarthen, 2010 were inspiring reading), especially psychotherapy, and to explore if there are some deeper layers contributing to empathy and the experience of being understood which is so elementary in psychotherapy.

My proposal is to combine CA and a special part of embodiment theory taken from CL (Johnson, 1987; Lakoff, 1987). Lakoff's subtitle (1987) was “What categories reveal about the mind.” Huge parts of this influential book refer to cognitive theory and experimental psychology, especially to prototype theory of Eleanor Rosch (Rosch and Lloyd, 1978) and her followers (Varela et al., 1993). In CL metaphors are no longer viewed as part of “texts” but as part of cognitive operations. They organize one's understanding of the world and of oneself-being-in-the-world. Metaphors have the potential to generate surprising kinds of category type and content (Glucksberg, 2008). In preverbal children's play the creation of metaphors can be detected (Tomasello, 2008). Metaphors become an element of thinking and not only of speaking.

In CA there is a deep interest in categorizing. CA is not only about “turn-taking,” repair activities etc., but also about “doing categorizing” (Sacks, 1980; Lepper, 2000; Schegloff, 2007a,b). However, interestingly enough, there is no mentioning neither of “categorizing” nor “metaphor” in the topic index of “The handbook of conversation analysis” (Sidnell and Stivers, 2013).

However, both influential traditions, CA and CL, deal with categorizing activity as part of human cognition and conversation. Embodiment is a useful thread to combine both with the aim to come closer to a solution of the riddle how cognition is influenced by conversation. This means (a) to better understand what “understanding” means in clinical practice, (b) how it is done, (c) how empathy is co-organized by two embodied participants. Empathy is a practice of “doing empathy,” not a magical or mystical equipment, it is no one-way endeavor. However, both participants indispensably use categories and they use conversation in order to match the difference. The *musical* dimension operates in leveling differences bearable. Sometimes, there is “groove” in good therapy sessions. However, this should not lead one to overlook many *musical* dimensions of conversationally “doing empathy.” Empathy can be studied as embodied practice in clinical contexts.

In CL it is assumed that all categories used to organize one's experience can be derived from bodily sensory experience. Thus, the Cartesian assumption of a mind in the body can be reverted to that there is a body in the mind (Johnson, 1987). For example, the prelanguage bodily experience of “balance” can be shown to apply to mathematical equations (Lakoff and Nunez, 2000; Nunez, 2008, 2011) and high levels of abstract mathematical thinking. Combining CA and CL might 1 day arrive at a subtle understanding how (therapeutic) conversation can influence the cognitive “apparatus” of clients enmeshed in seemingly unresolvable difficulties. In a CA+CL-approach categories care for “order at every point” as talk-in-interaction does. The ambivalent question (Heritage, 1984) like “Why don't you come and see me sometimes?” can be heard as friendly invitation or as reproach. The answer will inform the questioner about how the second speaker categorized. The change of category is an important aspect of therapeutic change—often achieved by therapist's using reformulations (Antaki, 2008). The client metaphorically speaks about somebody about whom he “exploded like a volcano,” the therapist may ask: “What made you so indignant?”—Using another metaphor with the effect that the emotional event appears in a new frame. These examples may suffice here. Categories operate in a multidimensional way. One cannot do without. How can this approach be applied in the analysis of clinical conversations?

It was Harvey Sacks (1992, p. 117) who reminded us that to understand another person you must use the distinction between “observables” and “communication.” First, you look at another's bodily clothing and behavior, gesture, mimic display in her face, you are struck by a gaze and listen to her voice. Then, you conclude something from these observables, you categorize these observations as indices of social status, gender, race etc. *and* of an internal state like (generally) intentionality or joy, pleasure, shame or the like. Third, you begin to speak following the rules of a (local) culture. Fourth, while observing and categorizing you realize that the same happens to you. Fifth, a cycle of mutual observing and reasoning is created within a few moments and within this cycle, sixth, the therapeutic task is to generate conversational contributions smoothly urging the other person to “doing opening,” which means to give that kind of knowledge that makes a common production of “empathy” possible. In any case, there are embodied persons mutually observing, categorizing, producing utterances by bodily voices for bodily ears. (Cf. Reich, 2010). Doing conversation by embodied persons solves the old philosophical problem of how people often “do understand each other” although they cannot look into another's mind.

The CA+CL-approach has been theoretically detailed and empirically validated by an extended qualitative study of a 4-year group therapy with sexual offenders in prison (Buchholz et al., 2008; Mörtl et al., 2010). We studied a huge corpus of transcribed group therapy sessions. The CA+CL-approach proved useful to gain a better understanding of how these people talk. While overtly confessing what they had done they secretly allude with certain indices to arouse listeners in the group, they use and share askew metaphors and they skillfully exploit a therapist's authenticity to blind his understanding. Their cognitive apparatus uses a high level of empathy in a very instrumental way. It seemed useful to combine CA and CL in order to make these phenomena hearable and visible for our analyses. And in order to understand how two very skillful therapists managed to overcome these difficulties and to bring these men in a deeper examination of what they did which brought some of them into a serious suicidal crisis. If this crisis was passed we could observe that they had changed the use of metaphors, their way of talking and categorizing things.

This paper starts with CA and its utility for the analysis of studying empathy as embodied practice in psychotherapy. Embodiment concepts of CL and CA will be integrated. Transcribed examples will be analyzed. The second part reviews some findings from infant research, psychotherapy process research and conversation analysis in order to mine neighboring fields in the expectation to find some treasures for embodiment theory, gaining new kinds of data or methodological progress. This might contribute to gaining a clearer, empirically-based definition of what we mean by empathy—two embodied persons engaged in producing mutual understanding. The paper concludes with some ideas about further research.

## A combination of conversation analysis and cognitive linguistics: useful in studying empathy as embodied practice

The therapeutic relationship is seen as the most influential factor in psychotherapy. Several dimensions have been differentiated. Since Freud's distinction between a “decent” and “transferential” relationship dimension, most modern researchers and clinical therapists see the importance of a “working alliance” (WA). Several instruments have been developed to measure the degree of working alliance since Bordin (1979) inaugurated a tripolar theory differentiating a working alliance into “aims,” “tasks,” and the entire “bond.” This conception integrates the (disembodied) rational means-end-orientation (MEO) of psychotherapy with the emotional side of the relationship (ESR). Ruptures of working alliances typically show up in the MEO—patients do not comply with arrangements, dates, appear too late to a session or forget what they had agreed to—but most often have their origins in the ESR: a sensitive domain of their experience has been addressed in too rough a fashion, they feel criticized by the therapist or devalued. The contributions of the therapist to alliance ruptures cannot be ignored either, e.g., the therapist might try too intensively to explore negative feelings or was not “licensed” to do so. Colli and Lingiardi (2009, p. 721) propose to differentiate therapist's failure into relational (empathy, attunement, warmth) and technical (type or focus of intervention) failure. Thus, as Safran and Muran (2000, p. 165) have pointed out, alliance is not a static variable based on mutual agreement only (MEO), but emerges from *resonances* in ESR. Scales have been constructed to measure the collaborative interaction of the WA (Colli and Lingiardi, 2009) or how the alliance is (re-)negotiated after ruptures (Doran et al., 2012).

The details of verbal exchange are so meaningful that serious doubts arose whether therapeutic talk can be analyzed by pre-established codings (Stiles, 1988, 1995; Stiles and Shapiro, 1989). However, CA is a micro-analytic method with a finegrained methodological view for saving elusive data against a too strong theory. CA has its origins in Ethnomethodology and social linguistics and since has proved an enormous potential to discover new phenomena (Martinez et al., 2012). It can be combined with similar approaches like analysis of metaphor (Buchholz, 1996/2003; Cameron and Maslen, 2010). CL (Lakoff, 1987) has shown that metaphors are rooted in embodied experience. Metaphor is understood as a mapping from bodily experience into more abstract domains. In CL bodily experience is conceived of by a number of bodily schemata as *container, path, balance, force*. They are understood as organizers of bodily experience that map this experience into more abstract domains. One of the first proponents of this view was Johnson (1987) in a very influential attempt to overcome philosophical cartesianism. This line of reasoning was followed by publications viewing the body as the prominent organizer of human experience (Gallagher, 2005; Hari, 2007), even in therapeutic theory (e.g., Lombardi, 2008). Johnson (1987) conceptualized a “metaphorical projection” according to which bodily experiences of “containing” were “projected” in abstract domains as in sentences like “Let's go into this topic now”—the topic itself is formatted as a container and the proposal is to enter this container. Other sentences like “my future lies before me” project the bodily experience of moving in a physical space onto the construction of a “time as a path”-metaphor. The viewing of a relationship as “balanced” is directly taken from the toddler's experience of balancing one's body when standing up and learning to walk. A special part of Lakoff's theory of metaphor (Lakoff, 2008) details this mapping from neural theory via experienced embodiment into abstract thinking. Thus, a new theory of metaphor has emerged based in embodied experience (Glucksberg, 2008). This is paralleled by quite similar developments in CA.

This has consequences for the conception of empathy. Empathy over a long time has been thought as something an “empathizer” applies to the one “empathized.” Neisser (1980) wondered why so much experimentation and theorizing considered participants as “passive onlookers” (p. 603) interested in science-like theory-testing. Schlicht (2013) criticizes the methodological individualism of this thinking and, in parts, experimentation. In real-life it is important for me to understand the other person correctly and not only “test” my theory about the other's “theory.”

The rationalistic bias of MEO is to be overcome as it is two bodies talking-in-interaction. Neither cognition nor empathy is an individualistic endeavor. And conversation is not “verbal behavior” transmitting coded messages to a decoding receiver. This outdated terminology led research to technical MEO-orientations. What was overlooked is that there are bodies thinking, talking and constructing opportunities for empathy (and blocking).

CA is not only interested in a semantic dimension of talk, but more in the organizational level. When we follow this line of reasoning that “turn-taking” is the cradle of meaning (Schegloff, 1999) we can track the continuity from deeply embodied early infant proto-conversations to adult discourse on high levels of symbolic encounter. Sequencing, repair-activities, synchrony of gestures and a lot of other features is as present in adult as in mother-infant conversation (Braten, 2009). Embodiment-theory talks of “emergence” (Brinich, 1982; Varela, 1990; Colunga and Smith, 2003; Tschacher and Bergomi, 2011).

How do people manage not to interrupt each other all the time? How do they deal with “trouble” (if someone does not reciprocate greetings or does not answer questions)? How do they know what can be said and what not (e.g., telling a dirty joke, Sacks, 1978)? The overall assumption is “order-at-every-point” which means that talk entails orderliness which participants produce *and* use to make sense of their interaction continuously. To hesitate in responding, to accompany the other party's talk with “confirmation utterances” or information receipt tokens (hm, hm), to withdraw your gaze, or raising your voice for a moment become significant events in order to produce meaning of the interaction itself—by the participants. Astonishingly, voice as the embodied producer of meaning *per se* is seldom paid attention to neither in clinical nor in research papers in psychotherapy (see Weiste and Peräkylä, 2013, for an exception).

Talk-in-interaction is hardly imaginable without embodied voice. It is one of the most surprising things that all therapists' unavoidably most used tool, talking-using-voice, has hardly found any research interest in the therapeutic sphere. Conversation analysts (Streeck, 2011) observe in finegrained detail how bodily movements of hand, gaze and body posture in everyday conversation contributes to the organization of talking and understanding. To speak of “mindful hands” as Jürgen Streeck (2011) does, could in therapeutic contexts become complemented by an observation of “mindful voice”—clinicians know how a voice can calm and sooth, attack and heal, prepare a carpet of empathy in a dialog or make everything said unacceptable. The analysis of multimodal metaphor has begun to include bodily gestures and the analysis of voice (Forceville and Urios-Aparisi, 2009; Cienki and Müller, 2010) and this converges with more recent studies in CA. Here is an enormous potential for future studies.

Production *and* usage of orderliness is compatible with “construing in action” as we have learned since Daniel Stern's classic “Interpersonal world of the infant” and his following excursions into the moments of meeting between adults (Stern, 1985, 2004; Leitner, 2007; Cipolletta, 2013). I will comment more extensively on what baby observation and conversation analysts have in common.

CA approaches can best be understood by demonstrating how it is done. Up to now CA has hardly been applied to a huge amount of verbal data for statistical analysis. CA demonstrates results by extensively presenting verbal data in transcribed form and showing how these data can be analyzed when participants try to make sense of their interaction.

Thus, CA will be used here in order to analyze psychotherapeutic talk-in-interaction. It has been applied to a lot of areas (e.g., medical communication, conversation in court, laughter, repair activities, emotion talk etc.) and is now shown to reveal undiscovered aspects of psychotherapeutic activity (Peräkylä et al., 2008). Sidnell and Stivers (2013) provide an extensive introduction to the method (for a short look see my review of this volume, Buchholz, 2013). What we don't have is a full precise description of psychotherapy on a conversational level, although this task is begun (Peräkylä et al., 2008). Here the dynamic system aspect comes into view. My first example will show the ups and downs of a wave-like interactional exchange.

## Examples

### Example 1: waves of divergence and convergence

I take as a first approach a transcribed example from Thomä and Kächele (1994, chap. 4.1) where the patient Nora comes 5 min late to a session, something which is not normal for her. The emotional situation between her and the analyst is clearly described:

“When she finally arrived, I was surprised to see her smiling and beaming with happiness; upon entering the room, she looked at me longer than customary and in an inquisitive manner. Her happiness and my displeasure created a very discordant contrast.”

Then the dialog is transcribed^1^ as follows:

P: Well, what's actually preoccupying me, I think, was the last comment I made before I left, about paying, which was also the topic of the previous hour, and I just thought it was rather telling that precisely that same topic was the last point in the conversation I just had with my boyfriend, although initially we had talked about something entirely different.

We are informed that she quarreled with her boyfriend in a restaurant about paying for the coffee, a quarrel which she described as “a back-and-forth like in a ball game *(Hin und Her wie ein Ballspiel*).” Using this metaphor of a *ball game* she uses an embodied activity as the source of imagination and this becomes a metaphor for this therapeutic discourse.

She remarks that still something is going on. She wonders whether she might be happily smiling either because she had left her therapist waiting or because of the situation in the restaurant. The question of who pays for what, she muses, had been a topic in the last therapy session, too. So several contexts of “paying” are brought together here. The transcript continues:

P: Now today… I'll play it where it's about speaking my thoughts, holding them back, then with the bills and… I'm wondering whether there is a connection with my being late.
A: Hum, I'd think so.
P: You'd think so. Ok, so I take away time. It's just, I actually also divide it up differently, and so my boyfriend and I were together a little longer.
A: We recently spoke about you wanting to give a good whopping to your boyfriend, and today it's my turn.
P: Yeah, I enjoy it.
A: And that's why you were beaming at me like that when you came in.

The focus of attention shifts to an expansion of the metaphor of game and play affected with a waving air of amusement and fun. She then connects this with interactional scenes, but she does not yet take full responsibility as she speaks in a non-actor mode: “my being late” instead of “I came late”^2^. The analyst reacts in a diffuse manner, and his utterance is hedged by an alignment token: “hum,” he aligns with the patient's pleasure in the game.

Then, this game successfully rearranges itself in the interaction, the patient responds with “You'd think so” in a playful manner. Now she moves on in an actor's responsibility mode: “I take away time,” pre-announced by the compliance token “Ok.” To take time away is a cultural metaphor, as if time were a thing that can be taken away. Again, the source of this metaphor is an embodied experience (“take something away”). Regarding this, the therapist responds: “today it's my turn.” He responds within the metaphorically created domain of a ball game.

Here now the pleasure (“I enjoy it”) of “taking away something” appears on the scene. In the German original version the therapist uses a strong metaphor in a humorous way in response to the cheerful tone of the patient, namely “*überbraten*,” evoking the image of being whacked over the head with a frying pan, in order to describe what it is she seems to be taking pleasure in when interacting both with him and her boyfriend—a new self-description (instead of ball game) of what happens in this conversation appears taken from another embodied source.

What we find is a very complex move in order to empathize with the patient consisting of several steps: we see the embodied patterns of observing and being observed (see my introduction) when the therapist describes the study of the patient's face. Here we can focus on the ebb-and-flow waves of the conversation when these observations become part of the conversation. We see a) an alignment of the therapist; b) enrichment by other interactional scenes having the effect of a “go-ahead!” directed at the therapist; c) the therapist's response with another enrichment (“we recently spoke about…”); d) return to the initial observation when the patient came in; now this observation becomes a common object of conversation.

Although the therapist (emotionally) disaffiliates with the patient's being late he (conversationally) aligns with an expansion of metaphor and with “hum.” By the steps described a new way is paved toward a new shared conversational space. It is not only a new object (“being late”), but a new level of consideration for each others' concerns is co-established (i.e., the meaning of being late). In everyday conversation being late is expected to be a subject to reproach. Obviously, the therapist feels an inclination to this as we are informed in extra-conversational comments. But in his utterances during the conversation he manages to affiliate *and* to initiate a new level of conversational consideration.

The therapist then comments on his own participation:

“I [the therapist] shared my impression with the patient to make it clear to her how much she enjoyed coming late and how much pleasure she had acting out aggressive impulses.”

The therapist's initial anger at being left waiting (reported in the comments only) seems to be overruled by the patient's fun in this game. The patient finds a new metaphor for her pleasure letting the therapist wait. The transcript continues:

P: [Laughing] Honestly—and I think that's where the expression is coming from—this gives me a feeling of devilish pleasure.

The German phrase “diebisches Vergnügen” (lit. “thievish pleasure”) here should be understood as “fiendish (or devilish) pleasure.” Affect and behavior are now linked in this illicit-fiendish or forbidden pleasure; the patient's repelled aggression was expressed both in her pleasure and in the fact that her behavior for the moment had been at the expense of the relationship to the therapist.

A: Yes, that's clear, and you let yourself have this pleasure. But I'm not very sure whether you also see the consequences of your pleasure.
P: Yes, well, the question of “What do I get out of it?” I haven't so far asked myself yet. But when I raise it now, then I do think that acting this way I gain your attention, because you might think “What's keeping her?” or something of the sort, and then I also realize how I react if someone else is late. It actually annoys me a good bit.
A: Hum, you seem sure of that.
P: That it annoys me. But that it annoys others, I don't want to know too much about that.
A: Isn't just that the source of your pleasure, that you can get people quite upset in a seemingly innocent manner.

What we have here is the emergence of *equifinal meaning* (Donnellon, 1996). Two speakers starting from different points for “moments of meeting” (Stern, 2004) arrive at a plateau of consensual understanding that is quickly left again. This I call the *convergence of meaning*. In everyday talk most people assume that convergence of meaning is the ultimate goal of conversation. This is, obviously, one side of the communicative coin only. If meaning converges, conversation ends. Convergence of meaning cannot last long. It is achieved when patient and analyst agree that to let someone wait is an enjoyable pleasure.

**Figure 1:**
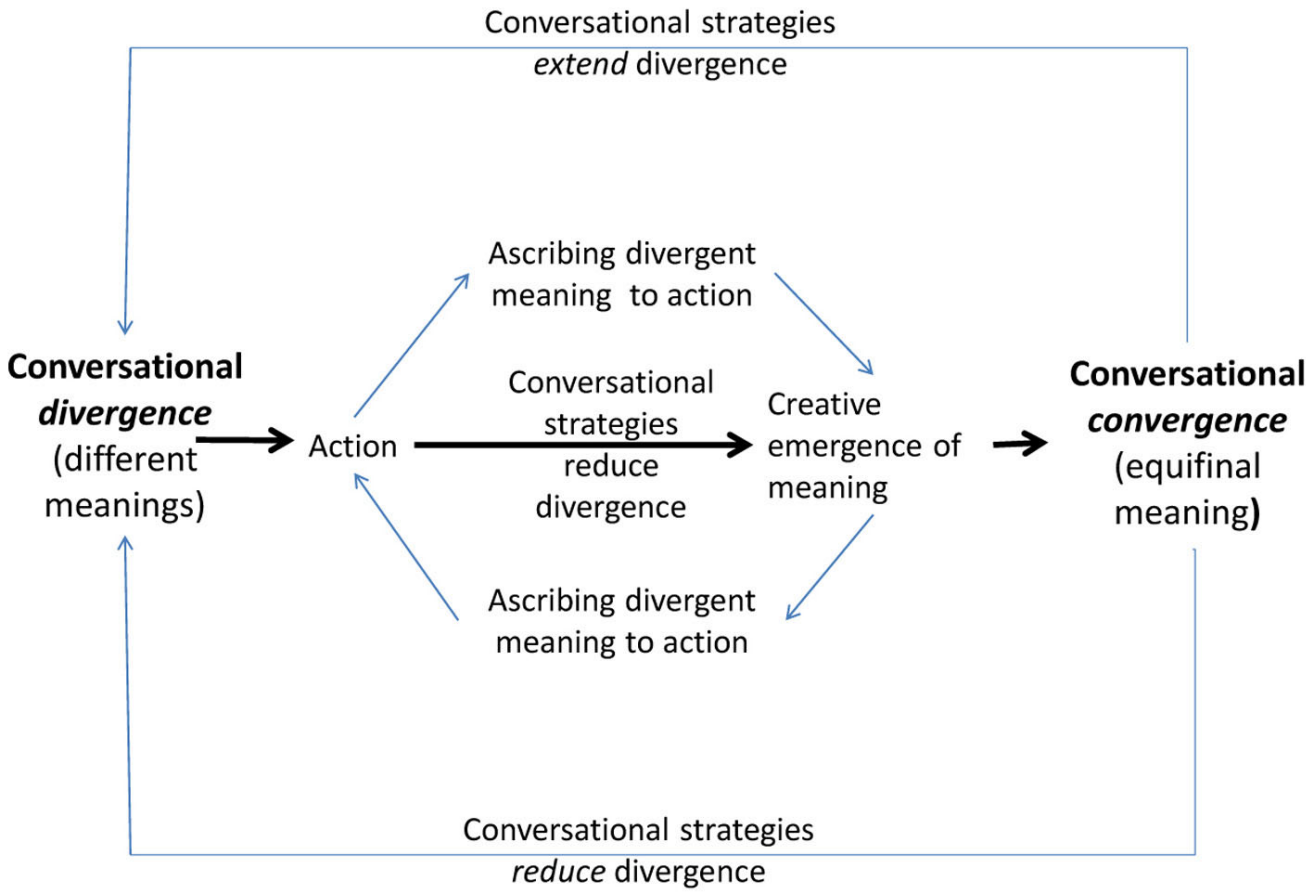
**Course of a conversational wave dance**.

Now a contrary maneuver starts. The therapist disturbs the convergence of meaning by *diverging* conversation; he says that today it is his turn. He is the one made upset as the patient violated the rules of the “ball game.” Ball games are ruled by mutuality, and who violates the rules is stopped, the other player can restart the game. This is embodied interaction although, on the level of verbal exchange, nothing seems to happen but talking. The rules of embodied interaction are absorbed into the more abstract domain of verbal exchange—and both speakers follow these embodied rules (see Figure 1).

This is the part the patient did not want to see (“I don't want to know too much about that”). Is there another interpretation possible that the patient is simply not concerned about her behavior's effects on others?^3^ I would object that the patient's overall readiness to debate the whole topic speaks against such a view. This readiness is communicated to the therapist and he can refer to it by diverging, which adds a new meaning to the conversation.

The conversational strategies used to produce equifinal meaning can be described as follows:

- Metaphors (e.g., game and play, “überbraten”) are used here in order to reconcile diverging opinions.
- Logical arguments: premise and conclusion are here used as rhetorical figures in order to mutually promote the other person's agreement.
- Modulation of affect: a sequence where one speaker starts with the expression of affect which is then either expanded upon or constrained by the next speaker.
- Linguistic indirectness: some use of passive constructions indicating some denial of agency, mitigators (“Hum, I think so,” “perhaps”), imprecise articulations which prove as helpful to continue conversation.

The conversation here begins with an affective discordant experience, conversation diverges: the therapist has no pleasure while he is waiting for his patient. The patient comes with a smile on her face—a maximum of divergence. Being late and the mutually recognized affect is the “action” that has to be debated. Convergence begins at this level of being prepared to commonly debate this divergence. Hereby certain conversational strategies reduce the affective disalignment.

Both participants use the conversational strategies described and add diverging semantic and, more importantly, affective meaning to how the “action” is to be understood. They finally achieve equifinal meaning, they converge^4^—and in the next move they start again to diverge from this point in order to begin a run through the same complex conversational pattern again. Convergence is just a momentary plateau of rest which is immediately given up in order to continue conversation.

This pattern cannot be reduced to properties of one of the participants or to a method “applied” by one participant to the next. One might say that a skillful therapist imagines this kind of experience as a kind of advanced organizer and strives to accomplish something like it, but the therapist cannot “make” it happen. What is needed is the special kind of empathic cooperation that can be described easily by *musical* metaphors: there is a wave-like rhythm of convergence and divergence between the two speakers, there are phases of affective resonance (differentiated in themselves between consonance and dissonance) and there is the aim to keep the relationship as a whole in balance, so that the cooperative work of meaning making can be continued. This constitutes a conceptual difference to what is called “alliance ruptures” because we can see here how the cooperative structure in general is maintained despite the divergence of meanings. The conversation as a whole is more a kind of play than an “exchange of information,” one can sense affective resonance even in the transcribed version.

There is a methodological difference between what psychotherapists *think* they do and what can be observed when they open their doors for scientific observation via audio or video recordings. Even in psychoanalysis it is becoming clear that self-description of professional work can only be one side of the coin (Canestri, 2011). The other side is “doing.” In interaction and conversation one cannot predict which “effect” an “intervention” will have, since this prediction, even if made silently, very quickly becomes part of the conversation and interaction and is responded to. There is no simple way “from cognition in one mind to conversation between two minds” (Potter, 1998; Coulter, 2005; Goodwin, 2007; Holt and Clift, 2007; Spurrett and Crowley, 2010; Deppermann, 2012). The methodological view used here originates from CA. As Harvey Sacks (1992, part I, p. 11) put it:

“When people start to analyze social phenomena, if it looks like things occur with the sort of immediacy we find in some of these exchanges, then, if you have to make an elaborate analysis of it—that is to say, show that they did something as involved as some of the things I have proposed—then you figure that they couldn't have thought that fast. I want to suggest that you have to forget that completely. Don't worry about how fast they're thinking. First of all, don't worry about whether they're thinking. Just try to come to terms with how it is that the thing comes off. Because you'll find that they can do these things.”

Surprisingly, one can find empirical evidence supporting this position. Similar to what conversation analyst Heritage (2002) described, in a study on “the pull of hostility” in therapeutic discourse (von der Lippe et al., 2008) a new design was created in order not to ascribe “hostility” to either the therapist or the patient. From a pool of 373 fully transcribed therapies 28 were selected. 14 Therapies were successful and 14 not. These cases had been treated by 14 therapists. From each therapist, one case with a successful outcome and one non-successful one were analyzed. In this way, neither positive nor negative outcome could be reduced to personal properties of the therapist. The transcribed sessions were analyzed with the “Structural Analysis of Social Behavior” (SASB, Benjamin, 1974, 1996, 2010) and the authors find

“that in successful therapy therapist and client follow each other, as in a dance (i.e., the overall balance of positive and negative affiliation can be predicted for one by knowing the other), while this harmony decreases over time in treatment failures” (p. 429)

There follows an important observation:

“It seemed to be in the dialogue itself that the constructive or unconstructive therapeutic climate was created.”

This is a helpful remark to bear in mind when looking at my next examples.

### Example 2: an interplay of resonance

Sometimes in therapeutic conversation therapists formulate their utterances with pre-announcements like “You think that… ” Linguistic observers of therapeutic discourse (Scarvaglieri, 2011a,b) found that many therapeutic utterances start with incomplete half-sentences in the format of “… that you felt loved” or “… that you thought this was an attack.” Therapeutic discourse here makes use of a strategy that can be observed in everyday contexts, too. But therapists seem to speak in this elliptic mode more often. Completing the other's utterance seems to be a means to achieve various aims: (a) to move oneself into resonance; (b) to propel conversation forward; and (c) to help the client to overcome assumed obstacles. Clients often react with signs of relief when they hear another speaker articulating thoughts they never dared to utter. The therapeutic relationship then is in resonance. The experience of being understood in most cases is felt to be helpful; sometimes it is also what clients fear.

What if this kind of intervention fails? I want to compare two different examples treated by different therapists. In the second example I will show how a skillful therapist managed to be allowed to articulate what he felt in resonance with his client in a first interview, as the therapist's utterances with pre-announcements like “You think… ” were eagerly accepted and followed by the client's responses.

Here I show how a therapist^5^ completes the utterances of the client in a seemingly skillful way, but the whole conversation quickly deteriorates into a turn-taking fight. I want to find out if therapists can be given hints how to discern such moments and react differently.

P: und das hat mir auch jetzt=im=nachhinein (.) sehr leid getan daß i:ch das
  and also now in retrospect (.) I felt very sorry that I
  nicht (.) äh geschafft *hab=die* Zeit dort (..) sinnvoll für mich zu gestalten
  didn't (.) uh manage to use my time over there (..) in a meaningful way for me
  oder (.) sprich ich mein (−) ich kannte ja da meine Freundin schon und
  or (.) say I mean (−) at that time I already knew my girlfriend and
  vorher hatte ich eine andere (lacht etwas) Freundin, so daß ich also immer
  before I had another (laughs a little) girlfriend, so that actually I always
  (..) da über's Wochenende heimgefahren bin? und au:ch, mit dem Ziel?
  went home there over the weekend? and also with the aim?
  °unter der Woche sehr viel° gelernt? oder sprich eben die Arbeit versucht
  °studied a lot during the week?° or let's say well tried to
  hab zu erledigen di::e (.) so=sein=muß=
  finish the work that (.) should be done
→T: = um=dann=a:m Samstag=[Sonntag,
  *in order to then Saturday [Sunday*,
P:                                           [=genau.
                                                *[exactly*.
T: = heimfahren zu können=um [da:nn
  be able to drive home, in order to [then
P                                                  [ja
                                                    *[yes*
T: nicht arbeiten zu müssen,=
  not to have to work
P: =ja genau! und das war irgendwo ein Stück wei::t ein Fehler? Wenn ich
  yes exactly! and that was somehow a mistake to a degree? If I
  je:tzt=zurück[schau
  now look [back
→T:    [und Sie denken jetzt (..) das Heimfahren haben Sie
             *[and you're thinking now (..) going home you*
  gemacht um nicht allein sein zu müssen.
  *did that so you wouldn't to have to be alone*.
P: ja! bestimmt mit auch.
  *yes! definitely also that*.
T: in ^*^2. (Ortsname)
  in ^*^2 (place name)
P: bestimmt mit auch. obwohl ich eigentlich ziemlich schnell Kontakt
  definitely also that. even though I was actually pretty fast
  gefunden hab gell,
  *in making contact wasn't I*,
T: ja,
  *yes*,

The student who came for therapeutic help because of some obsessive compulsive disturbance here talks about how he suffered from not being able to use his time meaningfully. He had a girlfriend at the time—but he still had to study and do his chores. Here it can be easily seen how therapist and client complement each other's utterances. They talk like one mouth speaking—probably another aspect of embodied talk-in-interaction not too often observed in everyday interactions. This goes so far that the therapist can say “and you're thinking now,” and the client accepts and agrees succinctly. CA (Schegloff, 2007a,b) has developed a way of describing turn-taking mechanisms according to which interruptions and overlaps are handled by participants as a kind of “trouble” demanding repair activities to restore the order of turn-taking. Remarkable, that these repair activities are not practiced here. In this first interview there are many passages in which therapist and client seem to be in this kind of affective resonance so that a new order of conversation emerges and the therapist can complement the client's utterances and vice versa. To be engrossed at the conversational surface makes the phenomenon of resonance appear. The following important aspect should be pointed out: at the point where this interplay of resonance takes place, the therapist takes himself back with the final “yes”—he moves back into the position of a listener, not of a participant with directing initiative.

### Example 3: failed resonance

My third example is from another therapy conducted by a female therapist with a male patient. I want to contrast these two examples in order to highlight some differences.

P: ja. (--) das hat sie ähm (--) aber das ich konnte ihr das irgendwie nicht (--)
  yes (- -) that she did um (- -) but that to her I somehow couldn't (-)
  das war schon berechtigt also das war jetzt nicht übertrieben oder so und
  that was actually justified well it wasn't excessive or so and
  sie hat auch nicht (--) sie hats mir verboten wie es Frauen verbieten
  she didn't either (- -) she forbade me to do it like women forbid things
  ((lacht)) das tut mir weh ich möcht das nicht ((lacht)) also ähm
  ((laughs)) that hurts I don't want that ((laughs)) so um
  → (15.0)
T: also die hat Angst dass sie sie verlieren könnte,
  *so she's afraid that she might lose you*,
→P: ja, (3.0) das äh (9.0) doch es ist irgendwie schon ja das hat sie
  yes, (3.0) that uh (9.0) yeah somehow actually yes she is
  (27.0)

For readers it will be difficult to understand what's the subject matter here. However, that is less important than the organizing turn taking activity: the client closes his first remark with a token indicating that *he wants to continue*. But the pause of 15 s disturbs the turn taking routine. On the semantic level the client's message did not come to a natural transitionally relevant point. On the performative level of turn taking the client indicates that *the next speaker might take the turn*. Thus, the client exposes the therapist to an interesting form of contradictory behavior. Should the therapist take the turn or not?

The therapist now starts his utterance with the same word the client ended and continues with what (the therapist thinks) the client might have intended to say. The client continues with an agreement token followed by a long pause (3 s), restarts to take the turn, pauses again (9 sec) and does not finish his sentence. Another example from the same session reveals that this is an interactional pattern between the two participants:

P: ja, das kann sein also das ist jetzt glaub ich noch zu kurz (---) um das
  yes, that could be well I think that's still a little too short (- -) to
  sagen zu können aber
  be able to say that but
  (6.0)
T: aber trotzdem könnte diese (---) war eben so mein Gedanke ob das nicht
  but nonetheless this could (- - -) I just had the thought if this isn't
  äh bei Anke ein bisschen ich will nicht sagen Angst macht aber doch
  uh for Anke a little I don't wanna say frightening but yes
  nicht nur nicht nur ähm Freude macht.
  *not only not only uh: enjoyable*.

Again the patients ends his contribution with a semantically open clause and a performative “long” pause of 6 s. This makes it unclear to the therapist whether this is a transitionally relevant point for turn taking or not. And, just as in the previous part, the therapist starts by taking over the client's last word. This pattern occurs several times during this session so that, 2 min later, we see the following escalation of “trouble” and turn-taking disorganization:

P: also ich versuche da keinerlei Rivalität rein zu bringen aber (---) äh ich
  so well I try not to bring any rivalry into it but (- -) uh I
  nehme das schon wahr wenn das von ihm so zum Beispiel mal ein
  do notice it if for example occasionally from his side
  bisschen kommt also (---) ich hab glaube ich das letzte mal erzählt von
  he does it a bit so (- - -) I think the last time I talked about
  vor zehn Tagen das Wochenende (---) da wo die beiden sehr stark
  the weekend from ten days ago (- - -) when the two of them very much
  ausgerastet sind so (---) äh
  threw a tantrum so (---) uh
  (4.0)
  da (--) hab ich schon so ein bisschen gedacht er will schon wissen was
  at that moment (- -) I did actually think a little like he does want to know what
  los ist oder er will irgendwie (6.0) ja gestern sind wir mit dem (--)
  is going on or he wants to somehow (6.0) yeah yesterday with the (-)
  gestern, vorgestern? gestern sind wir mit dem Auto äh zum Hockey
  yesterday, the day before? yesterday we drove to the hockey training by car
  gefahren weil die beiden jetzt auch mal gucken wollten und sind da mit
  because the two of them eventually wanted to have a look too and drove gefahren und (--) äh da hab ich saß ich vorne und hab den Arm um ^*^
  with me and (- -) uh I did I sat in the front and put my arm around ^*^
  (---) ähm Sitz gemacht und da kam von hinten so ein kleiner Klopfer also
  (- - -) um seat and then from behind a little knock came so
→ [()]
→T: [()]
→P: [()]
T:[()] gehn Sie weg von
  get away from
  meiner Frau
  my wife
P: nja, (2.0) äh also von ihm her sicherlich nicht bewusst sondern es war so
  hum (2.0) uh well surely not consciously on his part but it was like
  von ihm her so ne Art spielen () das
  on his part a kind of playing () that
  (3.0)
T: hm=hm,
  *hm=hm*,

This is an example where something does “not work,” obviously; the therapist tries to complete the patient's utterances and overrides the rules of turn-taking. Two observations serve to be mentioned: (a) after this happened several times during the session a kind of disorganization in turn taking makes repair activities relevant; (b) it is interesting that the patient announces this event in a metaphorical fashion before it happens (“I try not to bring any rivalry into it”).

The arrows mark the escalation of a “rivalry fight” for the right to take the turn. It was impossible to transcribe more precisely as there are simply sounds of starts and interrupting but not a single understandable word. Instead of continuing the other's thought as in Example 2, here we see an escalation of rivalry. This is a *term presented by the patient* itself. From an embodiment point of view one might reason that he sensed the rivalry in turn-taking in the paragraphs presented before. Turn-taking is the cradle of meaning. It is an embodied activity, using breath, a common focus of attention for an orderly sequence of interaction and energizing the body for the preparation of an interruption, when one wants to take the turn not only at transition relevant points. This bodily experience he termed “rivalry,” using a bodily experience of fighting. The embodied source domain again is projected into the more abstract domains.

In the first example one could see how the therapist manages to emotionally affiliate and conversationally align with the patient followed by the emergence of a new level of commonly focused attention. Here, in Example 3, the establishment of a new conversational level is not achieved. Emotional resonance is put out of use, turn taking is unbalanced and the rhythm of the talk is disorganized.

By further contrasting these examples we find the following results. Such processes cannot be considered to be therapeutic “failures” in the sense that a therapist does not want to help. The client disorganizes the transitionally relevant point in a characteristic manner: semantic non-continuation and long pauses effect a disturbance in the therapist's reactivity. How to respond? It is the best intentions of the therapist, namely to intervene helpfully, that contribute here to the unintended effect of disorganizing the talk. The transitionally relevant point is semantically unmarked but pragmatically offered—and so the therapist either by taking the turn or not, has a good chance of failing. Whichever way the therapist behaves, it could be considered as false. Often clients complain after such conversational episodes that the therapist has interrupted—but a close inspection of the record reveals that at one level at least turn taking was offered.

There is a third option for the therapist in order to escape this pragmatic paradox of false responding or false non-responding: to consider silence as a response. If after a while the client might complain about the silent therapist, one might describe one's own silence as politeness, waiting for the client to complete the sentence. And one could then ask what the client had in mind during the long pauses. Ruptures are a co-production of both sides, therapist and client alike (Safran and Muran, 2000; Lepper and Mergenthaler, 2007). The study of detailed transcriptions might help to identify and understand how to repair such ruptures. To pay attention to such turn-taking organization might become a part of training therapists.

### Example 4: repair and mind reading

My next example stems from an everyday talk observed and analyzed by Paul Drew (2005, p. 170). Conversation analysts have observed and widely demonstrated that conversation has a consensus preference. It is for example easy to accept an invitation for lunch but it is complicated to decline. “No” is an option that forces the participant with conditional relevance to provide a justification, an explanation, some kind of account why “not.” The *format*(put in square brackets) for an invitation refusal can be described by three conversation moves:

[Appreciation] + [(mitigated) Declination] + [Account]

When invited, a speaker will first respond with an appreciative remark, will then decline (more or less embedded in “softeners”) and will subsequently provide an account as to why it is impossible to accept the invitation. Here is an interesting example by Drew (2005, p. 170):

1. Emma: Wanna c'm do:wn 'av [a bah:ta] lunch w]ith me?=
2. Nancy: [°It's js] ()°]
3. Emma: =Ah gut s'm beer'n stu:ff,
4. (0.3)
5. Nancy: Wul yer ril sweet hon: uh:m
6. (.)
7. Emma: [Or d'y]ou'av] sup'n [else ° ()°
8. Nancy: [L e t] I:] hu. [n:No: I haf to: uh call Roul's
  mother, h I told'er I:'d call'er this morning...

Emma invites Nancy to come down “and have lunch with me” and while talking Nancy does not interrupt her. There is an overlap (indicated by the brackets [and]): she is immediately starting her (mitigated) refusal (line 2) calmly (indicated by °) but very early, too. The calm voice is an embodied aspect of conversation here; it indicates Nancy's very early awareness of the whole format and more, that her declination might hurt Emma and be followed by a change of state of her relationship.

Emma quickly (indicated by =) adds an attractive offer that she has “got some beer and stuff” (line 3) followed by a delay (line 4). Then follows Nancy's “appreciation” (line 5) addressed not to the invitation but to the person of her friend Emma. Then we have a short pause at a transition relevant point. Emma takes the turn offering “or do you have something else” (line 7).

This is the interesting point here. Emma offers an alternative account for Nancy's refusal and Drew here makes an important comment:

“This is a ‘cognitive moment,’ in a double sense: in order to make that move, before Nancy makes explicit her declination, Emma has to have *realized* that Nancy might be going to decline her invitation; she thereby *reads Nancy's mind*, attributing that *intention* to her.” (Drew, 2005, p. 170)

Drew wants to point out here that “intention” is not only a philosophical term but a practice performed by conversational participants in order to ascribe motivation^6^. In order to understand the process of mind-reading addressed here it is, of course, not necessary to assume telepathic abilities. Their voices have indicated declination and some repair activities. This observation can be extended to the assumption that both participants have an unconscious knowledge of the standard format how to refuse invitations among friends. This format is instantiated when Nancy in line 2 calmly begins to speak, followed by Emma's offer of “beer'n stuff.” This must not be viewed as an intentional pressure on Nancy to come. It is an alternative account for the refusal Emma has sensed embodied with Nancy starting to speak in line 2. There is a common format steering this conversation and, of course, this format is determined by a shared culture not by cognition-in-one-mind (Cerulo, 2002; Miller, 2006). Part of these cultural practices is to organize conversation and talk around eating. It is this cultural habit that allows Emma to anticipate Nancy's declination and try to get ahead by offering something better for the body. Several aspects of embodiment (calm voice, attractive dinner and eating) and the use of distributed conversation formats functionally operate together.

This example has something in common with my next one. In the Emma-Nancy interaction, Nancy's offer in line 5 can be seen as a “third move.” The first one is the invitation, the second one is the positive or negative response. The refusal, as Drew's analysis shows, is anticipated by Nancy's remark in line 2 and so Emma can insert the offer of line 5 in order to gain more acceptance for her invitation. Following a first (calmly spoken) resonance, Emma adjusts her invitation and this adjustment is influenced by Nancy's calm utterance. As Peräkylä (2010) has been able to show, this immediate correction procedure is just what happens in psychoanalytic therapy.

Braten (2009) shows how repair activities begin in preverbal mother child interaction. Some of these repairs anticipate the other's (negative) state (a mother feeling that her baby's body feels pain when handled in a certain way) and react to events that can, but must not happen. This feeling-the-other's-body is an embodied precursor of later conversational repair activities. There is “doing empathy” in conversational repairs.

It is very inspiring to read how conversation analysts (Corrin, 2010) observe very similar patterns in early child talk with their mothers. Helping and being-helped, asking and receiving an answer, greeting and being greeted, smiling and smiling back are examples for complete interactive patterns children acquire in early childhood. These patterns are expanded with the advent of language acquisition, then, they form expectations of how others should behave. And this continues in everyday conversation between adults. Perhaps this might be, what Gregory Bateson (1961) thought of in his notion of the “pattern connecting” diverging minds.

### Example 5: The “third move” after an Interpretation

Annsi Peräkylä is professor for microsociology in Helsinki and in addition a well-trained psychoanalyst. He has begun to publish a series of papers (Peräkylä, 2004, 2005, 2008, 2011, 2013) on how psychoanalytic talk is being conducted beyond theoretical self-descriptions of psychoanalytic colleagues.

Peräkylä (2010) used a corpus of 58 audio-recorded psychoanalytic sessions involving two experienced psychoanalysts and 3 patients. Here is one of his examples. The female patient has a partner who is seriously ill, the patient takes care of him and talks about her experiences and feelings:

1. P: As I had a dream that I had been (0.6) quite
2. insane for a few days.
3. (0.8)
4. T: Yea[h.
5. P: [so as one (0.4) as as one probably wants to
6. (1.8) empathize the (1.6) patient in his (1.1)
7. condition and his situation and and one wants to go
8. along with it, (0.4) then (2.9) one goes too far.
9. (6.2)
10. T: Probably in the same time you also repress the.hhh the
11. immense grief that [(arises) from that]
12. P: [Yeah. then it is is] is ((clears throat))
13. hh impossible.hhhh ((coughs)) to erm deal (0.2)
14. really with (0.6) that grief because (.) one has to act
15. all the time.
16. (0.4)
17. P: Or to be rational and to do dusting and to
18. order (0.5) things from pharmacy and (0.8) and this and that.
19. (2.2)
20. T: And on the other hand (0.6) you can also avoid the grief
21. through this very action.
22. (1.5)
23. T: So that it works b- [both ways]
24. P: [Yes, but as there is the]
25. responsibility so it erm some [kind of act] ion has to be
26. T: [yes]
27. P: acc[omplished.]
28. T: [It] has to be done necessarily.

The patient actively connects her being insane in her dream with her partner's insanity, proposing that this kind of excessive identification might reflect some kind of insanity on her behalf, too (line 9: “one goes too far,” which is a metaphor using an embodied experience). This prepares the way for the analyst's activity—Empathy cannot be thought as an “intervention” by an especially gifted empathizing person “into” a less able person; empathy here is clearly co-produced by both participants.

Obviously the body is present here, when the patient clears throat after her grief has been addressed by the therapist. And she uses bodily activities to (dusting) to avoid feeling sad.

The analyst addresses her active repression of grief when so intensely identifying with her partner. While first agreeing with “yeah” (line 12), the patient “then hastily moves into elaboration” (lines 12 ff.). But there is a difference. While the patient actively repressed her grief and tears, she talks about having “to act all the time” (lines 14 and 15). Again, she uses her bodily activities in order not to feel what the therapist tries to address. This is followed by a transitionally relevant point (line 16), where the analyst could have taken the turn, but keeps silent and so the patient continues talking about dusting etc. (lines 17 ff.). From line 20 onwards the analyst makes another interpretive turn. Peräyklä thinks, the therapist proposes

“a new perspective by pointing out that focusing on practicalities can also be a means for avoiding the grief. So, while ‘not grieving’ appeared in the patient's elaboration as something imposed upon the patient by the imperatives of the situation, in the analyst's third interpretative turn, avoidance of grief appears as the patient's own accomplishment, and the practicalities appear as instrumental in realizing this choice. By emphasizing the patient's agency or choice in ‘not grieving,’ the analyst also returns to an aspect of his initial interpretation where he suggested that the patient represses her grief.” (p. 1378)

By starting with “on the other hand,” the analyst combines both accepting the patient's perspective and adding a new one.

“The combination of the acceptance of the patient's elaboration and the explicit perspective shift is also embodied in the way in which the analyst, after the initial non-response by patient (line 22), pursues his suggestion in line 23. By pointing out that ‘so that it works both ways,’ the analyst suggests that both are true: that the patient is unable to grieve due to her responsibilities (perspective in the patient's elaboration) and that the patient uses her responsibilities to avoid grieving (perspective in the analyst's third interpretative turn).” (Peräkylä, 2010, p. 1378)

The artful operation of changing somebody's perspective is here skillfully handled by the turn-initial phrase “on the other hand”—again an embodied metaphor. Therapists of every kind use similar phrases without ever paying special attention as to how to initiate such an operation. But they do it successfully—by using implicit cultural knowledge of how to respect their client's view, unconsciously referring to formats like in example 4 and 5, and sometimes these operations open up a conversation to deep levels of common empathizing (Example 2), while at other times they fail, as in Example 3. Therapeutic conversation might be based on formats of that kind—more than ever thought. Therapeutic empathic skillfulness seems to be based in embodied knowledge of how to use common cultural formats with respect to a patient's topics and at the same time they hurt the patient's expectations, they violate rules of conduct, don't show respect for practicing avoiding feelings and then there is a skillful readjustment and fine attunement as repair activity of one's own utterances. This skillful handling of repair activities should be studied with greater attention. I assume it is based in early experiences of embodied repair activities during infant proto-conversation. There is continuity form proto-conversation to higher levels of conversation in using repairs.

In what follows I will turn to some evidence that could be used in psychotherapy process research. This evidence is presented here in the format of outlining some interdisciplinary lines of research especially between infant observers and conversation analysts.

## “Empathy is back”

Examples 1, 3, and 5 might give an impression as to how easily these complex therapeutic operations might fall apart. Ruptures in emotional resonance might give birth to violent escalations. Resonance, rhythm and balance should be considered relevant dimensions of empathic interaction in psychotherapy. In psychotherapy process research, the stage of understanding therapeutic “interventions” as technological procedures to be applied independent of the therapist-as-person should be overcome. In their influential work Orlinsky and Ronnestad(2005, p. 5) observed:

“As a rule, the study of psychotherapies has been favored over the study of psychotherapists—as if therapists, when properly trained, are more or less interchangeable.”

They see this attentive paucity as founded in a modernistic MEO-bias which led to the assumption that not the person of the therapist but the standards of method, procedure and technique are responsible for success or failure of the psychotherapeutic endeavor—this is an example of disembodied thinking viewed as a failure of research orientation. These assumptions fall in line with scientific standards of rationality and objectivity. What was termed *personal equation* from early astronomy (when astronomers looked through telescopes their observations deviated a little bit from one another) should be ruled out as a source of error which was to be controlled by experimental design. This disembodied kind of scientific understanding led to an elimination of the personal experience on the therapist's side, and one may wonder how this dimension should be brought back into the process by a therapist trained in this kind of reasoning only. Huge amounts of money were spent to research therapies—as if they could be conducted independent of the therapist. The NIMH study on depression conducted sophisticated statistical analyses to decide this question, and it looks so far as if the “therapist effects” had made the race (Elkin et al., 2006, 2007; Wampold and Bolt, 2006, 2007; for an overview of this debate see Buchholz and Gödde, 2012). It seems as if we meet a kind of paradox here: within the statistical area of objective science its counterpart, the embodied therapist's personal element and subjectivity, reappears. Since the publication of these results, many researchers have turned their attention to the therapist.

This development is accompanied by a reappearance of empathy in other areas than neurological research of mirror neurons. I leave out this topic as so many others have written about it with greater competence than I have. However, there are parallels in philosophy (Batson, 2011). Karsten Stueber (2006), a German philosopher teaching in the United States for many years, is well informed about the debates in German philosophy at the beginning of the 20th century. Prominent names are Theodore Lipps, who was highly respected by Freud; Max Scheler, who gave “Einfühlung” a central position in his philosophy; Friedrich Theodor Vischer, who saw empathic qualities as a presupposition for moral reasoning with respect to the different world views of others. Their opponents, such as the founders of the Vienna Circle, in those days were representatives of a more rationalistic philosophy in many variants. Stueber accomplishes a heroic task. He considers all the debates taking place at the time, brings the opposing positions of the prominent philosophers into a dialog and manages to cite a lot of empirical research. Prominent here is, of course, research on mirror neurons, the debate between simulation theory and theory-of-mind-theory, the role of folk psychology and cultural contextuality as opposed to explanatory approaches. In Stueber's analysis, MEO-conceptions of empathy tend to misunderstand empathy as a kind of theoretical enterprise, the body is missed in his analysis, too. Philosophers from the Wittgensteinian and hermeneutic tradition agree that empathy cannot be conceptualized as an analog of theory. So they replace the concept of empathy by “understanding” and this is mostly fixed to a textual level (Stueber, 2006, p. 195).

Stueber comes to define the limits of empathy (see also Breyer, 2013) not by rationality; in his view it is folk-theoretical conceptions that cannot sufficiently differentiate between correct empathic perceptions and prejudicial forms of understanding. Thus, certain constraints are to be acknowledged as in the case of different cultures. Here, further cognitive strategies are to be supplemented. But without empathic utterances everyday social interaction would break down in seconds. This holds even more for therapeutic conversation. Conversation, as I hope my examples have demonstrated, is more than just the exchange of propositions or mutual information about states in the world. Conversation includes the body.

### Knowing and feeling (of the other's knowing)

The earlier opposition of *naturalistic* experimentation as the “hard” version and *hermeneutic* understanding as the “soft” version of practicing science is outdated. As philosopher Wolfgang Detel (2011) states, in the current situation, perhaps surprisingly, strong impulses for a mutual rapprochement come from recent experimental evidence. I will give a line of experimental examples relevant for the topic of embodiment here.

What follows is some linguistic experiments that aim at exploring the psychological environment of certainty or uncertainty that a speaker has when responding to a knowledge question. These epistemic shades of gray envelop the content of the answer and help the hearer decide how certain or uncertain the speaker is with his answer. This look into the other's mind is a link to empathy. The disjunction between empathy and propositions is here bridged as researchers take into account that what is important is not only the information given, but the *embodied person* making the utterance, or respectively the *relationship of propositional knowledge (information) and personal certainty or uncertainty*.

Speakers indicate the degree of epistemic certainty of a proposition with hedges, e.g., by introducing their utterance with phrases like “I mean” or “as far as I know,” using adverbs “anyhow,” “probably,” employing modality (instead of saying: “this is so and so” they say “It might be that… ”), and through changes in prosody (e.g., intonation, rhythm, and quality of voice). Not only knowing exists, but a “feeling of knowing” (FOK), as Hart (1965) termed it.

The assessment of the (un)certainty of an utterance can follow a procedure of counting the linguistic indicators here mentioned in a question-answer design (Smith and Clark, 1993). This design was expanded upon when the researcher's interest turned to “feeling of another's knowing” (FOAK), as in Brennan and Williams (1995). These authors found that listeners use a lot of resources to approximately assess a speaker's (un)certainty: (a) use of one's own embodied knowledge as a measure; (b) the assessed degree of question difficulty for the speaker as a tool to judge confidence; (c) the degree of mutual knowledge; (d) how the speaker is known or said to have performed in other environments previously; and (e) linguistic surface features, such as (f) latency to respond, (g) intonation, (h) forms of avoiding an answer etc. We find voice, hesitation, avoiding as embodied measures perceived by the listener with sensitive ears. Krahmer and Swerts (2005) turned to childrens' ability to detect (un)certainty in videos. While adults care a lot about uncertainty and, when uncertain, will increase frequency of pause production, display a higher pitch of voice, change intonation, lift their eyebrows and display an increase in smiling responses (embodied responses), children don't appear to care too much about uncertainty. Self-presentation, these authors conclude, is a less important thing for children than for adults.

Dral et al. (2011) used textual markers vs. prosodic markers to assess (un)certainty. They were looking for the possibility to automatically detect (un)certainty by prosodic markers. As for textual markers they differentiate hedges into different types such as “shields” and “approximators.” “Shields” obviously are designed to prevent failures and approximators are used as a politeness strategy in the flow of conversation. This is context-dependent, participants differentiate between these two. Embodied variables of prosody such as intonation, latency of response, intensity of voice and speed of talking were also put under scrutiny. They conducted several statistical analyses on a dataset of 552 audio files and in comparing the transcripts became optimistic that “(un)certainty in spoken dialogs can be assessed automatically.” The authors display a certain degree of optimism here. They find that to approximate (un)certainty “the textual features obviously score best” (p. 76). To a (unexpectedly) high degree, uncertainty comes in the guise of assessment or suggestion.

These empirical and experimental results can be taken as cues for the increasing attention given to the relevance of embodiment-dimensions in linguistics, in psychotherapy process research and in conversation analysis, too. New empathy-related questions appear: not only what is spoken about (informational content), but also who is speaking (the speaker's “identity,” see Antaki and Widdicombe, 1998), to what (recognized) contexts a speaker responds (“situatedness”), to whom someone is talking (recipient design, see Hepburn and Potter, 2011; Hitzler, 2013) and the positioning of the body in the physical room and dimension and the personal positioning in metaphorical descriptions for interaction (e.g., ball game) seem to me to be relevant dimensions for studying empathy.

As empathy like love can hardly be defined propositionally it is advisable to follow the strategy of conversation analysts here. These researchers don't use pre-defined concepts to be applied onto empirical data. They look for naturalistic data (Mondada, 2013) and study the various *practices* of empathy. The research question is not ontologically directed to what empathy “is” but to how empathy is “made” by participants in talk-in-interaction. Empathy emerges as a co-production. Heritage (2011, 2012) and Heritage and Lindström (2012), analyzing transcripts of everyday interactions found that articulation formats of empathy can be described. Following Goffman (1978) they analyzed “response cries” like “Oh!” and “Ah:h!” by which people embodied the expression of surprise, silent participation or follow the emotional paths of up- and downgrading excitements. Response cries (Hepburn and Potter, 2012) of that kind are articulated when you hear something you know: how a tooth was extracted, the first kiss or that somebody died.

Other conversational activities can be assigned to a “spectrum” (Heritage, 2011, p. 164) of empathic responses. “Ancillary questions” are uttered when another empathic response to a story told could be expected and the recipient of a story utters a kind of related question expressing some affiliative engagement with the teller. Ancillary questions have the power to refocus the matter in a way the teller could not have expected. The recipient thus opens a way to escape further conversational obligations in a single move. Often a teller cannot decide from ancillary questions how empathically engaged the recipient is.

This is different with “parallel assessments.” Respondents “can focus on focal elements of the experience described by the teller, by describing a similar, but particularized, experience or preference” (Heritage, 2011, p. 168). Someone praises the asparagus pie prepared by Jeff and the respondent utters something like “I love it. °Yeah I love tha:t.” He takes a “my side”-response in Heritage's term. There is a dilemma emerging in this kind of empathic affiliation with others:

“On the one hand, the recipient has not had direct first-hand experience of the event reported, and a parallel ‘my side’ response risks being heard as flat, pallid or pro forma. On the other hand, a parallel assessment that is too florid, extended or enriched in detail … risks being heard as competitive with the very report that it is designed to affiliate with.” (Heritage, 2011, p. 169)

Parallel assessment is a term to express the experience of “I know how this feels” or “I felt like you.” Heritage's goes beyond this and shows what a risky stuff lies in this kind of responding. Responding that way might be perceived as a contest about who might enjoy the privilege to continue telling. In therapeutic contexts the most conventional form of parallel assessments might be utterances like “I know that, too,” “yes, I do understand that” or the compliance token “hm:hm.”

On a higher level of empathic responses he finds what he terms as “subjunctive assessment.”

“With the term *subjunctive assessments*, I mean to introduce efforts at empathic affiliation which suggest that if the recipient were to experience the things described they would feel the same way.” (p. 169)

Think of someone telling of a wonderful meal and then changing the receipt in a certain respect and the recipient responds with “Oh yeah! This would be fantastic!” He never has eaten the meal prepared in this way but he answers in advance as if he would have; thus, presenting an evaluation of this experience in the conditional. Both use their body (mouth) to simulate an experience they have not yet had—and affiliate on that. The subjunctive mode expresses a time mode of futurum II, something that has not yet happened is treated as if it happened and can be evaluated as something in the past. In therapeutic contexts subjunctive assessments might appear when talking about a clients wish-fulfillents and future aims.

Heritage offers a further level:

“By ‘observer responses,’ I mean to indicate responses in which recipients claim imaginary access to the events and experiences described, but position themselves as observers, or would-be observers, to the event.” (P. 171)

With this format a listener takes the position of an imaginary or belated witness. In therapeutic contexts this might often happen when treating traumatized patients who suffer from having experiences nobody saw or listened to and thus are threatened by derealiziation of their experience.

Applying this schema of empathic response spectrum Kächele and Buchholz (2013) showed that in an emergency SMS-therapy different empathic reactions could be differentiated in their effects to the client. But it would be a serious error to assume that such formats of empathic responses could be deliberately “applied” in order to achieve certain effects in the other person. This is misunderstanding of the whole thinking and approach. No, what researchers find is the opposite of deliberate application. It “just happens” that people react that way. They have a feeling for what reactions and answers fit into the situation and in contexts. This feeling is a resonance evoked by such situations and contexts and only après coup this can be analyzed by a scientific observer. Being in resonance, Heritage finds in his further contributions is complemented by a balance of knowledge between participants. Someone mentioning a name never used before will immediately recognize if the person referred to is not known to the listener. He has a “knowledge surplus” (K+), the listener lacks this person reference (K-). Heritage (2012) speculates that to equalize this epistemic difference is a mighty impetus why people are in interaction. So the first speaker who realizes that “Peter” is unknown to the listener will immediately add the relevant knowledge (“Peter, my neighbor at the left side”) and knowledge difference is equalized. Balance is one of the embodied schemata Johnson (1987) and Lakoff (1987) have proposed as a source domain for so many target domains—this conception is applied here when Heritage describes how the knowledge difference is restored. Balancing is part of the universal cooperative structure of conversation (Grice, 1975; Tomasello, 2008). Affiliation is increased on both sides by such inobtrusive means.

Interestingly, this small conversational operation cannot be repeated. If someone in the same conversation were to add a second “Peter, my neighbor at the left side” this would show that here, too, is the potential for risky stuff. The same conversational operation is never the same. This contributes to the conviction that conversational contributions cannot be thought of as applicables. In the FOK and FOAK-terminology one could say this would hurt the balance of knowing and feeling between, at least, two participants. This reasoning makes plausible that balancing is a genuine embodied source domain that cannot be replaced easily.

### Toward a definition of empathy

The most surprising result seems to be that people employ all these dimensions, react via different channels (textual or prosodic, eye gaze or affective facial displays) and integrate them at high speed. In most instances of everyday experience this suffices to achieve sufficiently reliable conclusions about trustworthiness of conversational partners. This type of research results supports a view that transcends neurological and information processing approaches, where it does not suffice to correlate experience and the activity of the brain. Correlational thinking would simply accentuate the gap between what a neurologist and what a psychologist studies. *Empathy is the vehicle by which human beings, with their basically embodied enormous capacity for coordination, integration and organized movement, can create their own environment with respect to the other's state, context and situatedness*. The single organism's helplessness can be overcome by these abilities. From this evolutionary moment empathy alters environmental morphostasis to environmental morphogenesis which we perceive as cultural plasticity. Culture never is a single individual's product alone. To quote Whitehead (1929, p. 1):

“Culture is activity of thought, and receptiveness to beauty and humane feelings. Scraps of information have nothing to do with it.”

Culture has more to do with “feelings of knowing” and “feelings of the other's knowing” than with knowing/information alone. Infant researcher Colwyn Trevarthen (2003, p. 76) stresses this difference with great emphasis and points to a perceived error of research activity emerging from ignorance of this difference:

“In Edinburgh our Computational Linguists seem to have lost all interest in communication. The study of grammar cannot progress that way. It just gets more and more complex. But, as soon as you start to relate grammar to these spontaneous rhythmic characteristics of mother and baby communicating, then grammatical syntax gains a new meaning, a vitality and usefulness. What we are studying is dynamic emotional syntax; phrases and narrative sequences of feeling that are certainly foundational for the structure of verbal sentences, and their messages.”

Endowed with *dynamic emotional syntax*(DES), human beings, designed for culture, *must* be experts in empathy. This conclusion seems less disturbing when I add the constraints: as long as friendliness and a lack of serious conflict prevails. Conflict, trouble, quarrel, animosity and finally violence either let empathy disappear or instrumentalize it differently: in order to find out where the enemy can be struck the most harmful blow. In infancy, DES is constituted of rhythms of those “motives and emotions for actions that sustain *human intersubjectivity”*(Trevarthen, 2011, p. 121). Rhythm here means moving one's body in rhythmic synchrony with the baby's body. From rhythmic forms of shared emotionality, the ability to mutually read intentions emerges as well as the “flow of emerging self-awareness” (Trevarthen, 2011, p. 121). “To live as an animal, or as an infant … is to move with *good purpose;* that is, to want to act in ways that will be felt to be beneficial to the whole individual.” (p. 122).

### The turn to alterocentrism: concordant and complementary forms of empathy

What we generalize as “human relatedness” can be understood as a rich system of various patterns with an I-pole and a You-pole organized by rhythm, balance and resonance based in evolutionary view on mirror neurons (Ferrari and Gallese, 2007) and then advancing to conversational and symbolic organization. The whole system is built bottom-up, but if once established it operates more and more top-down. There are two dimensions in this conception: (a) a methodological problem of separating and integrating channels of empathy; (b) a developmental/evolutionary dimension. What follows are these two points of view.

To do empirical research in “empathy” seems to become a paradoxical endeavor. Empathy “happens,” it cannot be “applied,” it cannot be elicited by command and sometimes you have it, sometimes not. All researchers know this very well. So they cannot but try to establish an experimental copy of real world empathy. Battles and Berman (2012) direct their attention to what they call “conversational acknowledgers” presenting a list of 30 exemplars. This is very similar to what Heritage described as a spectrum from response cries to observer response. Such acknowledgers can be actualized on a verbal and nonverbal level (e.g., “hmhm” and “nodding”). Four experienced and licensed therapists of an eclectic orientation produced videos with pseudo clients. The hypothesis was that an increase of acknowledgers would increase the perceived empathy of therapists. The videos were produced in different fashion: some with verbal and nonverbal acknowledgers in a high degree, some with both low and some videos with a mixture. The videos were rated by 320 participants. The results show an unexpected consistency effect for both types of acknowledgers: if the rate of acknowledgers is equal (both low or both high) than empathy was rated high; if the video observers found an inconsistency than empathy-ratings decreased.

“Inconsistency in levels of verbal acknowledgers and nodding leads to miscommunication and perceptions of deception and sarcasm.” (p. 6)

is the author's best explanation. So what the 320 video observers observed was not only the sensory-embodied information: viewing with eyes frequency of nodding or hearing with ears the frequency of verbal acknowledgers. What they observed is a difference, an imbalance (again: balance as an embodied schema) between the two types of acknowledgers and their empathy assessment was dependent on this difference. *Observers go beyond sensory information*, they immediately conclude from “observables” to “invisibles”—a “difference” cannot be observed. In empathy assessment they use sensory information for immediate integration onto a higher level of meta-information concerning the integrity of the person emitting the sensory information. This is the way a top-down strategy begins to operate in the whole system.

The study group of Regenbogen et al. (2012) highlighted this topic by a complicated experimental design. They produced videos, too. They directed their research interest to the question which influence single channels of information have when observers attribute empathy to a story heard and seen in the video. Three channels were varied: facial expression of a story teller, the prosody of the voice telling and the content of the narrative told. The self-related stories, presented by well-trained actors, presented disgusted, fearful, happy, sad, or neutral narrative content. Four experimental conditions were varied. The same emotional expressions on the prosodic or facial expression level were added so that one could hear a story with neutral content, but with a sad voice and happy facial expressions. Or in the “neutral condition” all channels were equal. A complex variation of channel combination led to the production of 64 short video clips which were to assessed by 40 healthy persons. They had to rate the emotions seen on the video and the emotions observed in themselves while attending to the video. Some other measures such as Galvanic skin reaction were taken, too. The statistical analysis of this complex design again and again presents “a significant main effect of Communication” (p. 1002, p. 1003, p. 1004, p. 1005). This phrase was repeated again and again; it refers to the match of self and target emotion, to the matching of self and other, to perceived naturalness, to intensity levels, to a comparison of non-empathic and neutral responses etc.

Another important result was that once a channel was neutralized, the number of empathic responses decreased. Speech content was particularly involved for assessing the participants' own emotion.

“Based on these findings, we attribute a specific role to speech content, contrary to several findings previously suggested… During the appraisal of several-channel information, neutralized speech might represent a context in which the bimodal emotional information cannot be integrated. In other words, the neutral speech content did not give a rational explanation of the emotional visual-auditory perception and subsequently might have inhibited an emotional perspective change, which is a prerequisite for empathy.” (p. 1009)

And the authors make this point very clear:

“Summarised, the present findings suggest that in human communication, behavioural empathy relies on consistent information from several sources; facial expressions, prosody, and speech content. Omitting one channel generally results in decreased empathy judgements, lower intensities and fewer psychophysiological reactions. The requirements for empathy are differently affected by different communication channels. While missing emotional information in the face decreases a person's ability to recognise someone else's emotional expression displayed by other channels, omitting emotional information in the speech content causes the largest drop in performance rates of adequate emotional reactions.” (p. 1011)

Cautiously, we can assume this to be experimental evidence for the influence of the conversational dimension including sensory-embodied perception which gradually increases in development and allows for a top-down influence in empathic perception.

Now I want to turn to the developmental dimension. It is interesting to observe that some prominent experimental researchers also do not hesitate to quote some of those authors philosopher Stueber quoted. Frans de Waal (2007) contributes a “Russian doll-model” of empathy in an evolutionary perspective. De Waal turns against cognitive conceptions of empathy. Empathy and theory of mind (ToM) cannot be set equal. Autism in children cannot be explained by a ToM deficit because autism can be found before the age of 4 years. But what are the antecedents of ToM? The answer is in de Waal's view that “at the core of the empathic capacity is a relatively simple mechanism that provides an observer (the ‘subject’) with access to the subjective state of another (the ‘object’) through the subject's own neural and bodily representations. When the subject attends to the object's state, the subject's neural representations of similar states are automatically activated. The closer and more similar subject and object, the more perceiving the object will activate matching peripheral motor and autonomic responses in the subject (e.g., changes in heart rate, skin conductance, facial expression, body posture). This activation allows the subject to get ‘under the skin’ of the object, sharing its feelings and needs, which in turn foster sympathy, compassion, and helping.” (de Waal, 2007, p. 59).

This automatic embodied response is called the “Perception-Action Mechanism” (PAM), a description that fits well with other authors', such as Damasio's hypothesis of emotions (2010). What is important here is not only the close match between contemporary authors' theorizing. Going further, de Waal hints at Lipps who, long before modern neuropsychological research, pointed out that what he called *Einfühlung* meant something like “feeling into” by which he meant something like inner mimicry. Empathy is a bodily re-construction of the object's state of mind, feelings and other inner states. “Accounts of empathy as a higher cognitive process neglect such gut-level reactions, which are far too rapid to be under conscious control,” de Waal (p. 59) adds.

There is a neural basis of empathy as matching the other's state, leading to emotional contagion. This process is based on PAM using motor mimicry and matching the actions of others, as in the case of yawning or imitating other people's gestures. This provides the base for higher levels of cooperation and shared intentions leading to cognitive empathy and understanding the other's need for help. At a third level the other is actively imitated and his/her state is emulated, thus attributing certain emotions and mental states and generating a difference between “my” and “your” perspective. This difference reiterates the process of individuation while empathy creates bonds. So empathy is a phenomenon which “covers a wide range of emotional linkage patterns, from the very simple and automatic to the very sophisticated” (de Waal, 2007, p. 62).

Here I would like to suggest a differentiation between concordant and complementary empathy.

If a chimpanzee mother sees her baby unable to come down from a tree, she will reach out her hand to give just that kind of support and help the baby needs. If a human mother sees her baby fighting with a woolen blanket that is too warm, she will remove it with a gesture and talk soothingly to her baby. This is complementary empathy: both figures involved, the baby and the mother, have a different stance. One can offer the kind of help or support the other needs and is willing to grant it. Different qualities and different perspectives complement each other to form a circle of helping-helped-relationship. Concordant empathy can be differentiated from that: if you hear a poem that moves your heart you react in concordance, re-constructing automatically the same state within yourself as (you think) the poet expressed in the poem. Here, not difference but equality of emotion is the main factor.

Both forms of empathy are guided by what Tronick (2007) termed a “dyadic state of consciousness.” Tronick aims to conceptualize the experience of being included in a “higher” level of functioning if together with someone else and if both are attuned at the same wavelength. There is no sender-receiver-relationship, this is rather included in a part-whole-relationship constituting new forms of experience, personal identity and emotional quality.

“This dyadic state organization has more components—the infant and the mother—than the infant's (or mother's) own self-organized state. Thus, this dyadic System contains more information and is more complex and coherent than either the infant's (or the mother's) endogenous State of consciousness alone. When infant and mother mutually create this dyadic state—when they become components of a dyadic system—both fulfill the first principle of systems theory of gaining greater complexity and coherence. The gesturing mother-held-infant performs an action—gesturing—that is an emergent property of the dyadic System that would not and could not occur unless the infant and mother were related to each other as components of a single dyadic system.” (Tronick, 2007, p. 407)

Tronicks thinks that entry into this kind of dyadic state of consciousness is an indispensable precondition for psychotherapeutic help. A client must have a feeling that the therapist knows in his own body what kind of pain the client feels and that the therapist's actions are guided by this “feeling into” the client's emergency—and this embodied base of empathy must be communicated. The feeling of being in this state of good care prepares the client to endure the stress and strains that are bound to come up during the course of psychotherapy. Empathy can be considered a means to achieve this indispensable *dyadic state of consciousness*.

This state is indispensable not only during infancy. An adult suffering from toothache will endure the necessary surgery much easier when feeling that the doctor is in resonance with him/her and will conduct the treatment with care and compassion. A child at school age who failed in an exam may accept help with studying only when she experiences some form of emotional resonance from her teacher. Someone with a broken leg from an accident lying on the ground will accept the risk of painful transportation only if he can feel that the emergency assistant does what must be done with a sensibility for what might cause the patient harm or pain. A psychotic on a psychiatric ward who is in a fit of rage because of a feeling of being treated with injustice will calm down when someone tells him convincingly that he is understood in his claims. Resonance is an indispensable experience.

## Conclusion

In the first section I presented conversational data with the intention to point out some embodied phenomena of empathy. I have tried to bring together CL and CA by using the theory of embodied schemata Lakoff (1987) proposed. Although these areas are still somewhat strange to each other as reflected in their positioning to “cognition” (Potter, 1998; Deppermann, 2012; Potter and Edwards, 2013) they seem to converge with respect to embodiment. From embodied schemata there is a direct path to categorization which is object of study in CA and CL. Thus, certain aspects of embodiment concepts can help to bring theories of cognition and conversation closer together. To include the body as an interactional resource of meaning generation is of highest relevance for studying therapeutic process and for therapeutic listening as an embodied practice. The way of analysis proposed here followed this convergence into the domain of therapeutic “talk-in-interaction.” To include the body does not necessarily involve video-recording only. The body is present in talk—and this can be made “hearable” in audio-recording and transcripts following the convergence of CL and CA as outlined here. Of course, a lot of empirical research has to be done in the future.

My analysis showed repeating waves of divergence and convergence in a therapeutic dialog, a second and third example showed the quality of mind reading evolving in one and failing in the other conversation, the fourth example compared this with an everyday practice of anticipating the other's next move and the fifth example from a psychoanalytic session showed how the analyst adjusted his interpretative activity following the patient's response to a first interpretation. The embodiment aspects have been outlined in all these examples. In these examples one can see how sensitive conversation is based in being aware of the of the other's body (voice, moves to prevent being hurt, repair activities), how it operates in talking and how this sensitivity is done, practiced by analyzable moves, turn taking units, anticipations. People think, respond and react to thinking people and they know that these people are thinking how they themselves think about what's going on while things go on. Empathy should no longer be conceptualized as a special “tool” gifted persons (therapists) use; empathy should be conceptualized as a coproduction of both embodied participants using embodied mindful voices. New research questions arise: what activities are brought into conversation to prepare the cooperative coproduction of empathy? How is cooperation in this special domain prepared and organized? The answers might be found in the direction of moving waves between convergence and divergence, of making use of metaphors, of speaking rhythms, prosody and the like—aspects that can be understood more easily when abstaining from disembodied concepts of mind and conversation.

In the second section I tried to organize a theoretical tour d'horizon along some recent linguistic, philosophical and conversational studies available when one studies empathy. Philosophers turn to empathy again and they refer to neuroscientific studies reporting results which in part have been well formulated in former years by philosophers without having neuroscientific evidence at hand. There is astonishing evidence between these areas of research and new formulated theories from infant research. Further research of empathy should include the continuity of conversation from embodied early childhood proto-conversation to high levels of abstract communication, the overall cooperative organization of conversation which in early years can be described by *musical* metaphors but which are not lost when high levels of communication are achieved. Balance, rhythm and resonance (Buchholz and Gödde, 2013) on high levels play their embodied role in order to make empathic understanding possible from embodied proto-conversation stages of development onwards. Further psychotherapy process research should more thoroughly consider this musical dimension of embodied conversation. Process research can make use of detailed transcripts in order to detect these dimensions.

Finally, some remarks about how empathy ruptures might risk giving birth to conversational forms of violence. Conversational violence has been shown in a qualitative interview study with 90 participants (Buchholz and von Kleist, 1997) as a consequence of failed empathy. False empathic understanding has serious consequences for the “empathizer,” too. If co-embodied empathy operates it creates a sense of being-in-contact fulfilling a deep human desire. Yet, increasing numbers of ruptures of the empathic bond are responded with some kind of withdrawal, aggression or, at least, verbal violence. This is felt in a certain place: in the therapist's body.

### Conflict of interest statement

The authors declare that the research was conducted in the absence of any commercial or financial relationships that could be construed as a potential conflict of interest.

## Footnotes

^1^Timo Buchholz (based on the English-Version of Thomä and Kächele vol. II) retranslated the transcript-parts from German into English.

^2^This distinction is not meant as a linguistic one; in German the difference is stronger than in English. In any case it is the responding analyst treating this difference as relevant.

^3^As an unknown reviewer helpfully asked.

^4^My terms of convergence and divergence have something in common with the more general terms of affiliation and disaffiliation (Muntigl et al., 2013). These terms set the accent to the emotional experience, my terms to the conversational exchange making visible the wave movement of conversation here (Thanks to an unknown reviewer for this hint).

^5^Many thanks to Horst Kächele, Berlin, for the audio record I used for transcription. Pauses are indicated by (.) for a length of less than 0.2 seconds; (..) is a pause of 0.2–0.4 s and (−) is a pause of 0.5–0.9 s length; numbers in brackets, e.g. (2) indicate a pause of 2 s. Quiet speaking phases are marked by °… °. Latching is indicated by = which means that no discernible silence between two turns could be detected. Lengthened syllables are indicated by a colon:. Overlaps are indicate by [. This is a very reduced version of transcription rules normally used in CA-papers. For a full description of the CA approach to transcription see Hepburn and Bolden (2013).

^6^Potter and Edwards (2013) refer to this example in order to convincingly demonstrate that “intention” must be considered a mundane term; “intention” is not something to explain conversational practice. It is used by participants themselves as “account.”
